# The Miracle of Mercy

**DOI:** 10.1093/ojls/gqab017

**Published:** 2021-05-23

**Authors:** Giordana Campagna

**Keywords:** mercy, pardons, rule of law, treating like cases alike, state of exception

## Abstract

Many states governed by the rule of law provide for mercy powers, most prominently the power to grant pardons. This raises a potential conflict: the rule of law requires that like cases be treated alike. Mercy, on the other hand, is usually understood as being unfettered by legal principles, including the principle of treating like cases alike. Focusing on state officials’ pardon powers as an example of mercy, this article argues that we should conceive of pardons as miracles. Like miracles which suspend individual laws of nature, pardons suspend legal rules on an individual and discretionary basis. Once suspended, the legal rules no longer apply to the given case, and consequently there is no conflict between pardons and the rule of law. As I will show, this discretionary suspension of legal rules is a prerequisite for pardons to fulfil their function as a corrective to undesirable legal results.

## *1.*Introduction

In 1999, Harold and Dewayne Damper, two brothers from Mississippi, received the same sentence for a crime they committed together, and for which they were indicted and tried together. Both brothers served their sentences at the same prison in the Southern District of Mississippi. Fifteen years into their sentences, both petitioned President Obama to grant them a pardon. Dewayne’s petition was granted. But Harold’s was not.^1^

Apart from its obvious unfairness, this case raises a broader legal problem. The two brothers’ cases were alike in all relevant respects,^2^ yet they were treated differently. This seems to be in direct conflict with one of the rule of law’s cornerstone principles: treating like cases alike. If a president or other state official shows mercy to A, it would appear that he is obligated to show mercy to B, if their cases are alike in all relevant respects. Yet in the Dampers’ case, one brother walked free but the other did not. Although this is a particularly jarring example, it points to a more general tension that befalls all exercises of mercy in rule of law states. In contrast to the rule of law, which requires that like cases be treated alike, mercy (of which pardons are an example) is usually understood as being unbound by rules. Mercy, it is often argued, is like a gift whose giver is not constrained by any rules—including the rule of law. Hence, if we required mercy-givers to apply mercy according to the requirements of the rule of law (equally to all like cases), they would cease to be mercy-givers. Mercy and the rule of law thus appear to be in conflict.

In this article, I argue that this conflict can be resolved if we understand mercy as a *miracle*. My claim will be that pardons in particular are isolated abrogations of legal rules. Once abrogated, legal rules no longer apply to the given situation, and the conflict between pardons and the rule of law disappears. The main idea is that the rule of law requires cases that are similar to be treated by the law in the same way, so that once pardons have done away with law there are no longer any concerns about the rule of law. The analogy with miracles is particularly fitting, as in both cases—miracles and pardons—the ordinary course of an event is set aside by a greater power.

I will develop my argument as follows. In section 2, I will lay the groundwork of my argument by shedding light on the ostensible conflict that many authors claim arises between mercy and the rule of law.^3^ To do so, I will first provide an account of mercy, highlighting that discretion is one of the central features of any plausible conception of mercy. I will then contrast this with the rule of law, by focusing in particular on the principle of treating like cases alike, which is contained in all conceptions of the rule of law. The section will end by showing that the discretionary element in mercy is considered to be in conflict with the rule of law’s principle of treating like cases alike (I will refer to this as the Conflict Thesis). Section 3 will rebut the Conflict Thesis with regard to pardons. I will argue that pardons can be regarded as compatible with the rule of law if we conceive of them as miracles. My argument will proceed in four steps. First, I will draw on Austin Sarat and Nasser Hussain’s concept of ‘lawful lawlessness’ to show that pardons are best viewed as provided for by law, but not regulated by it. Pardons, in other words, are ‘alegal’. I will apply this idea to the Conflict Thesis to show that pardons do not violate the rule of law even when they treat like cases differently. Secondly, I will argue that, *pace* Sarat and Hussain, the ‘alegal’ character of pardons bears more similarity to miracles than to a state of exception in Carl Schmitt’s sense. Thirdly, I will demonstrate that the exact point of pardons is that they are like miracles, serving as a last resort in cases where the normal application of law has yielded undesirable results. Finally, I will argue that the increasing scrutiny in legal systems towards pardon powers does not necessarily lead to the demise of the miracle of mercy. The Conclusion will retrace the article’s main steps.

One word of clarification before I begin. The ‘alegal’ nature of pardons does not render them immune to criticism, for while, as I argue, they are not subject to legal assessment, they can nevertheless be evaluated on non-legal grounds. For example, pardons could still be challenged on the ground that they violate the requirement to treat like cases alike, as is found in most accounts of justice.^4^ My argument is, more simply, that pardons cannot be challenged on the grounds that they violate the version of this requirement which is based solely on the rule of law.

## *2.* Mercy and the Rule of Law

To understand what I call the ‘miracle of mercy’, it is first necessary to get a better sense of the conflict that many believe arises between mercy and the rule of law. In this section, I will first give a brief overview of the concept of mercy, before I will turn to outlining the rule of law’s principle of treating like cases alike (A). I will then (B) show why the two are regarded as being in conflict with each other (Conflict Thesis).

### *A.* The Concept of Mercy and the Rule of Law

It is not an easy task to define mercy. Even a quick glimpse into the literature reveals that there is no generally accepted concept of mercy. Instead, there are a whole range of different conceptions that are partly congruent and partly contradictory. This article does not aim at spilling any more ink on this subject. Instead, I propose to focus on Alex Tuckness and John M Parrish’s thorough examination of various conceptions and metaphors of mercy. In their book, Tuckness and Parrish identify three common characteristics of mercy, which I will adopt for the purposes of this article.

First, mercy ‘presupposes[s] some form of inequality of power’.^5^ This means that I can only be merciful to you if I have the power to make your situation better or worse. Secondly, ‘characteristically, mercy involves helping or benefitting the person in an unequal position’.^6^ In other words, I only show mercy to you if I choose to treat you leniently instead of harshly. Thirdly, ‘the agent showing mercy must have, in some sense, discretion about whether to bestow it’.^7^ Put simply, if I was forced to treat you leniently instead of harshly, I would not show mercy to you.

The third element of mercy—its discretionary character—is the most important one for our purposes. Although there is some disagreement as to what exactly this entails, there is a general consensus that mercy is discretionary. This idea can be found, among other places, in the popular metaphor that mercy is a ‘gift’. This is usually taken to mean that mercy is ‘*freely* given’,^8^ that it is ‘unconstrained’^9^ or ‘an act of grace’.^10^ To treat someone mercifully is thus to treat them more leniently than one in fact *could* have done.^11^

In legal terms, to say that mercy-givers have discretion—that they are free (not) to show mercy—is to say that they do not have a legal *obligation* to bestow mercy.^12^ An act of mercy is therefore never one which one is obligated to perform. The mercy-giver must be unconstrained by law so as to be able to freely choose whether or not to grant mercy to the mercy-receiver, and thus whether or not to benefit the person in the less powerful position. The other side of the coin is that no one has a *right* to receive mercy from the mercy-giver.^13^ Mercy is not something one is entitled to; it is something one begs for.^14^

And this, precisely, appears to pose a serious problem when we are dealing with cases in which it appears that someone would have a *right* to be treated equally to others whose circumstances are sufficiently alike.^15^ This right follows from a cornerstone principle of virtually all rule of law accounts: the principle that like cases ought to be treated alike.^16^ The consistent endorsement of that principle is striking given the considerable disagreement that exists on how the rule of law should otherwise be conceptualised.^17^ As Brian Tamanaha points out, there are three common themes that run through all conceptions of the rule of law. One of these themes is ‘formal legality’, which, as Tamanaha notes, ‘emphasises a rule-bound order established and maintained by government’.^18^ Concretely, this means that laws must be public and prospective and have ‘the qualities of generality, equality of application, and certainty’.^19^ As Tamanaha explains elsewhere, the requirement of ‘equality of application’ is the requirement that the laws be applied ‘equally to everyone according to their provisions’.^20^

This requirement is widely recognised by rule of law scholars, but often with different terminology. Lon Fuller, for example, proposes that the rule of law consists of eight ‘principles of legality’,^21^ one of which is the principle of ‘congruence’.^22^ According to Fuller, congruence requires that how the law is applied actually conforms to what the law on the book says. That is, it requires that the laws be applied equally in conformity with their terms. Andrei Marmor presents yet another way of referring to this notion of legality. He uses the term ‘consistent application’ to describe the requirement that there should be ‘considerable congruence between the rules promulgated and their actual application to specific cases’.^23^ ‘[F]or the law to function properly,’ Marmor argues, ‘its promulgated rules must be the rules which are actually applied to specific cases by the various law enforcement agencies.’^24^

Regardless of the terminology used, the requirement of congruence has an important implication: it entails a requirement that like cases be treated alike—and a corresponding right to equal treatment. If a rule requires a certain outcome in a particular set of circumstances, then whenever the same (or an alike) set of circumstances arises, the same rule must be applied consistently and therefore the same outcome is required (alike treatment). This is why, for instance, Ben Johnson and Richard Jordan argue that the principle of congruence—or, as they call it, ‘consistency’—and the principle of treating like cases alike are in fact the same principle. ‘[C]onsistency is strictly pointless,’ they argue, because ‘it only enjoins theorists to apply the rules when they apply.’^25^

This line of reasoning leads to one further point. Some authors have argued that the necessity to treat alike cases alike follows not from a special feature of the rule of law—such as consistency—but from the idea of rule-following itself. Their reasoning is that rules are equally applicable to all cases falling within their scope simply in virtue of the fact that they are general rules. As Tamanaha notes, the rule of law’s ‘formal qualities [such as congruence] are *characteristic of rules as such*’.^26^ Similarly, according to Winston, the idea that like cases ought to be treated alike is already encapsulated in the idea of a government by law.^27^ HLA Hart put this thought as follows:

If we attach to a legal system the minimum meaning that it must consist of general rules—general both in the sense that they refer to courses of action, not single actions, and to multiplicities of men, not single individuals—this meaning connotes the principle of treating like cases alike, though the criteria of when cases are alike will be, so far, only the general elements specified in the rules.^28^

According to Hart, the principle of treating like cases alike means nothing more ‘than taking seriously the notion that what is to be applied to a multiplicity of different persons is the same general rule, undeflected by prejudice, interest, or caprice’.^29^ Hart’s point is that all cases falling under the same rule require the same outcome. Because rules make these legal consequences dependent on a certain state of affairs being the case, the same consequence is required whenever there is such a state of affairs. In other words, whenever two cases are alike in the relevant respect as determined by the rule, they ought to be treated alike. Hence, rules, in virtue of their characteristic of being rules, entail the principle that like cases be treated alike.

### B. A Potential Conflict

As I have outlined above, mercy is generally regarded as having a discretionary element which means that mercy-givers cannot have an obligation to apply their mercy, or to apply it in a certain way. The rule of law, on the other hand, confers on people in similar situations a right to be treated equally. With this in mind, I now turn to the Conflict Thesis, which holds that the two concepts are at discord with each other.

That there is a potential conflict is not a new insight. In 1077, the Benedictine monk Anselm of Canterbury raised a very similar point. In his *Proslogion*—a meditation on the existence of God—he asked: ‘if it can in some way be grasped why You [God] can will to save the wicked, it certainly cannot be understood by any reason why from those who are alike in wickedness You save some rather than others’.^30^ Anselm’s point was that it is difficult to explain how God can be merciful to some but not to others, yet still manage to be perfectly just.^31^

Anselm was, of course, interested in divine mercy and its relationship to justice, not in this-worldly mercy. It is true, of course, that human beings differ from God in that we do not lose what is characteristically human if we act mercifully but unjustly, or justly but unmercifully. That is simply to say that, unlike God, humans are fallible—very much so. But that does not mean that Anselm’s point is of no relevance to our secular world. Quite the opposite: the tension that Anselm discovered between mercy and the principle that like cases ought to be treated alike is still of great import today.

This point is illustrated by Ross Harrison.^32^ In contrast to Anselm, Harrison centres his analysis on the state rather than God. Harrison agrees with Anselm that, in principle, the entity in question (God for Anselm, the state for Harrison) should act mercifully as well as according to the principle of treating like cases alike. Hence, like Anselm, Harrison appears to have stumbled upon a dilemma that seems difficult, if not impossible, to resolve.

Harrison, who is one of the few philosophers to have committed themselves to discussing this problem in detail, frames his discussion of mercy and the principle of treating like cases alike not around two virtues, but around ‘four independently plausible, but mutually inconsistent propositions’.^33^ Harrison’s first proposition is that ‘states ought to be purely rational entities’.^34^ A ‘purely rational’ entity is defined as one whose acts are ‘justifiable’, which means that its acts ‘should be supported by reason’.^35^ Closely related to this point is the second proposition according to which ‘to act rationally is to act equitably, or (in one sense) justly’.^36^ A rational act is an act committed for reasons and ‘to act for reasons is to act on the basis of descriptions’.^37^ Crucially, Harrison thinks that descriptions—which are the basis for reasons—are ‘reapplicable’, arguing that ‘where the description reapplies, so does the appropriate act’.^38^ In short, he seems to mean by this that descriptions lead to reasons, which lead to acts. If the descriptions are similar, they lead to similar reasons, which in turn lead to similar acts. Harrison concludes that ‘therefore to act for reasons is to treat relatively similar cases in similar ways’, which is ‘what it is to act equitably, or (in one sense) justly’.^39^ Hence, for Harrison, to act rationally means to act equitably,^40^ which means to treat like cases alike.

Harrison then moves to the third and fourth propositions, which he considers to be closely related to each other. The third proposition is that ‘we want those in power over us (the state’s agents) to be merciful’.^41^ As is commonly believed, even ‘purely rational power’, such as that held by the state or God, should be exercised mercifully.^42^ The fourth and last proposition, finally, is more a conclusion than it is a proposition: ‘something that is purely rational cannot be merciful’.^43^ Harrison seems to claim here that to act mercifully is to act randomly. According to him, the state cannot act randomly because—unlike private persons—it ‘exists for the sake of the citizens’, for whom it must act rationally, not randomly.^44^ Based on his four propositions, Harrison reaches the conclusion that, when it comes to mercy, states cannot have their cake and eat it too; that is, they cannot act mercifully and equitably at the same time.

There is an intriguing parallel here with Anselm’s theory. It, too, is based on the insight that it seems to be impossible to be merciful *and* treat like cases alike at the same time. But Anselm reaches a different conclusion to Harrison. According to Anselm, God could, in principle, be just and merciful at the same time. This is because Anselm’s claim was based on his aim to prove that God exists. In a nutshell, he argued that things that only exist in our imagination are less perfect than things that exist in reality. Hence, because God is the most perfect of all beings, He must exist in reality. And part of being perfect is to combine all possible virtues, mercy and justice included. Realising the tension between a merciful and a just act, Anselm resorted to the following argument. The tension dissolves once we realise that the merciful act can be looked at from two perspectives: the perspective of the mercy-receiver, for whom the act appears to be at odds with what justice would have required; and the perspective of God Himself, for whom the act can still be considered just, because ‘it is befitting to [His] goodness’.^45^

Harrison, on the other hand, argues that, because the state cannot be merciful and respect the principle of treating like cases alike (ie be rational) at the same time, it should abandon its pursuit of mercy: ‘Only by forgoing mercy can we enable the state (or other authorities) to behave like a fully rational entity, accountable for all its actions to the people over whom it has power.’^46^ Only if states act rationally, Harrison suggests, does it become possible to review the actions of a state by reference to how well they conform with the existing rules. Put differently, states cannot be held accountable where they act mercifully rather than in accordance with the rule of law. For these reasons, Harrison concludes that ‘the state is not allowed an area of play lying beyond any possible justification. It is not allowed mercy.’^47^

As I will show in the section below, Harrison’s concern that mercy and the like treatment of like cases are incompatible is borne out in recent attempts to judicially review pardon decisions. For now, however, it is sufficient to take note of the conflict that has occupied many thinkers: the rule of law state and its officials need to respect the precepts of the rule of law in all their acts, and this requirement appears to conflict with mercy. As pointed out above, mercy is generally regarded to be free of constraints. Mercy is just like a gift whose giver is not bound by any rules as to whether to grant it or not. This means that if state officials were required to show mercy based on a certain rule, then their ‘mercy’ would not be unconstrained, which is the same as to say that it would not be mercy at all. State officials thus face a problem: they seem to be bound by principles requiring them to treat like cases alike, which appears to be in direct conflict with the discretionary nature of mercy. This is the Conflict Thesis. In what follows, I will argue against this Thesis and will show that, at least with respect to pardons, mercy and the rule of law do not conflict.

## 3. Pardons, Alegality and the Miracle of Mercy

In this section, I will focus on one particular instance of mercy—pardons—and will show that with respect to them, the element of discretion is not in conflict with the legal rule requiring that like cases be treated alike. This will strike many as counter-intuitive. *Prima facie*, it seems as though mercy powers would have to be treated the same way as other acts of state officials that are naturally governed by the rule of law. If, for instance, a president were to show mercy to A by pardoning him, it would seem to constitute a violation of the rule of law not to pardon B also, assuming that the cases of A and B are relevantly similar as a matter of law. However, this is not the case. True executive mercy *does* allow the official to treat A differently from B. One gets pardoned, the other hangs.

To explain this, I will argue in this section that the Conflict Thesis can be rebutted with respect to pardons once we recognise their ‘miraculous’ character. To put this in less mystical terms: pardons cannot conflict with the rule of law and its requirement to treat like cases alike because they are instances in which individual rules do not apply. I will develop my argument, first, by explaining executive mercy’s inherent lawlessness (or ‘alegality’). Applying this finding to the (apparent) conflict between pardons and the rule of law, I will show that pardons cannot violate the rule of law (A). Next, I will demonstrate that the lawlessness of pardons should be interpreted as a miracle rather than a state of exception in the Schmittian sense, as Sarat and Hussain have argued (B). I will support my finding by showing that the nature of pardons as miracles is essential to their function as a last resort in cases where the normal application of law would have undesirable results. Finally, I will address the potential demise of executive mercy caused by the increased judicial review of pardon decisions (C).

Before proceeding, I should note that I focus specifically on pardons issued by executive bodies. Depending on the form of a given state, executive bodies either constitute the sovereign or act on behalf of the sovereign (eg the people).^48^ For the purposes of my argument, the question of which role the executive bodies fulfil is not essential, as long as their actions can in a loose sense be attributed to the sovereign.^49^

### A. Lawful Lawlessness

To understand my argument, we first need to grasp what pardons are. In short, pardons are an institutionalised form of mercy. As such, they typically encompass all three elements of mercy which I outlined in section 2: they involve a beneficial treatment, an inequality of power, and discretion in choosing to either treat someone beneficially or not. The latter two features—inequality of power and discretion—are mirrored in the fact that pardons are a matter for sovereigns. That is, they are exercised by the sovereign or by officials acting on the sovereign’s behalf. Pardons are the business of kings and queens, of presidents and governors. The element of beneficial treatment, on the other hand, is reflected in the fact that pardons entail the waiving or mitigating of punishment.^50^ As I will show in this section, there are two aspects that make executive mercy special and set it apart from other acts of state officials: first, pardons abrogate legal rules, and, secondly, they are completely discretionary.^51^

I will start by considering the first aspect, that pardons set aside law.^52^ To understand this, it is important to see that while pardons are instances of mercy—the discretionary decision of a sovereign to alleviate or mitigate punishment—they are not just that: they also constitute a legal anomaly. This is because pardons abrogate individual legal rules for a given person and time. They are not exceptions to or amendments of the law. Rather, executive mercy relieves the defendant of all or some of the consequences of a conviction,^53^ thereby setting the law aside. As RS Downie puts it, ‘To pardon a person … is to let him off the merited consequences of his actions; it is to overlook what he has done and to treat him with indulgence’.^54^ Therefore, a pardon is not simply a decision as to whether, for instance, a prisoner walks free or not, although it is also that; a pardon is the significantly more momentous decision on the suspension of a legal rule for a given person and time. Pardoning someone is to say: the law requires you to serve 20 years in prison, but I decide that this law does not apply to you anymore. Hence, by granting a pardon, executive bodies break with the legal *status quo* as far as a specific rule (or specific rules) and a specific case is concerned. A pardon is law’s antithesis.

This aspect of pardons has led Sarat and Hussain to draw parallels between executive mercy and Carl Schmitt’s concept of the sovereign.^55^ According to Schmitt, the sovereign is the person or body that decides on the state of exception,^56^ which is the same as the state of lawlessness in Sarat and Hussain’s terms. The sovereign’s decision is neither lawful nor unlawful in the normal sense. Rather, it sets aside the legal order.^57^ As Giorgio Agamben puts it: ‘the state of exception is not a special kind of law (like the law of war); rather, insofar as it is a suspension of the juridical order itself, it defines law’s threshold’.^58^ According to Sarat and Hussain’s Schmittian reading, just as the law is set aside in a state of emergency, the law is set aside when a state official shows mercy by granting a pardon. In both cases, the law is overcome by a decision of the sovereign: by the body declaring the state of exception in one case, and by the state official showing mercy in the other. This decision, they argue, is different from ordinary decisions.^59^ In both ordinary and exceptional cases, officials have a choice. But the choice is of a different kind. In the case of ordinary discretion, the law allows officials to choose between outcomes a, b and c. In the case of pardons, by contrast, the law allows officials to choose that neither a, b nor c should apply.

There is, of course, an important difference between a Schmittian state of exception and the power to pardon. The former, as Schmitt points out, is ‘From the liberal constitutional point of view … no jurisdictional competence at all’.^60^ Rather, it is a political competence that transcends the legal order. As Schmitt argues, the ‘precise details of an emergency cannot be anticipated’, nor can ‘what may take place in such a case’ be spelled out.^61^ Regarding the state of exception, he claims that both ‘The precondition as well as the content of jurisdictional competence’ have to be unlimited.^62^

The case is different when it comes to the power to pardon, which is a competence provided for by law, even if the exercise of this power results in the suspension of the law. This paradoxical characteristic of pardons has been described by Sarat and Hussain as ‘lawful lawlessness’.^63^ The idea behind acts of lawful lawlessness is that the law can exempt certain acts from its ordinary requirements. Hence, legal systems can allow for powers—such as the power to pardon convicted criminals—that are not regulated by law. In these cases, the law enables lawlessness. Pardon powers are provided for in law itself and allow the bodies on whom the powers are conferred to freely decide whether and how they want to make use of this power in a concrete case. With Sarat and Hussain, we can thus say that pardon powers are a form of ‘legally sanctioned alegality’.^64^

Writing in the context of emergency powers, David Dyzenhaus has proposed along similar lines that the law has ‘holes’^65^ that are either ‘grey’ or ‘black’. A ‘grey hole’, according to Dyzenhaus, is a domain within the law that the law itself has created but that it governs only weakly. Dyzenhaus contrasts these ‘grey holes’ with ‘black holes’, which are completely unregulated by law, in much the same way that Schmitt conceives of the state of exception. As Dyzenhaus argues, ‘grey holes’ are in effect worse than ‘black holes’ because they confer a ‘facade of legality’ to official lawlessness.^66^ Using Dyzenhaus’s terminology, one could argue that pardons correspond to ‘grey holes’ and are problematic for the very same reason.

I will now turn to the second aspect of pardons: that they involve complete discretion on the official’s part. Pardons differ from ordinary discretionary powers in this sense. In contrast to the latter, which are bound by legal restrictions on how officials ought to exercise discretion, there are generally no restrictions on how officials exercise their pardon powers.^67^ Put differently, ordinary discretion powers are measured against the yardstick of law, but pardons usually are not. An act of executive mercy, as Sarat and Hussain observe, differs from ‘more regular instances [of discretion] since it can be an act of total discretion by a single executive officer’.^68^

To make the point about complete discretion clearer, consider the distinction between ‘duty-imposing’ and ‘power-conferring’ rules, which Hart has popularised. In a nutshell, duty-imposing rules require persons ‘to do or abstain from certain actions, whether they wish to or not’.^69^ Power-conferring rules, on the other hand, ‘provide that human beings may by doing or saying certain things introduce new [duties or obligations], extinguish or modify old ones, or in various ways determine their incidence or control their operations’.^70^ It is relatively easy to see that mercy powers belong to the latter category as they confer on their holders the power to establish or extinguish certain duties (such as the duty to serve one’s just sentence).

But do ordinary powers of discretion not confer on their holders a similar degree of leeway? After all, a judge might have it in his power to condemn someone to 20 or merely five years of imprisonment. To distinguish ordinary powers from pardon powers, we need to differentiate two different kinds of power-conferring rules. As Alf Ross has pointed out, power-conferring rules (which he calls ‘rules of competence’) ‘can be divided into two fundamentally different types’: private autonomy and public autonomy.^71^ One of the crucial differences between private and public autonomy lies in the degree of freedom the actor has in exercising the respective power. Private autonomy is a personal power that ‘is not tied up with a duty to exercise it, or to exercise it in a certain way only’.^72^ This contrasts with public autonomy, which is a power that ‘is not granted with a view to its being used freely by the competent person at his convenience’.^73^ To exercise public power is not a personal decision; it is an obligation. Philip Mullock, drawing on Ross, puts this point succinctly when stating that ‘the powers of officials are “duty-bound” and are not, as private powers are, granted with a view to their being exercised by competent persons as they see fit’.^74^ In other words, administrative acts are not acts that are freely undertaken, even if they involve discretion.^75^ Here, then, lies the difference between pardon powers and ordinary discretion powers: both involve a ‘discretionary’ element, but only mercy is dispensed freely. The nature of pardons is thus similar to what Ross calls private autonomy, whereas ordinary discretion falls into the category of public autonomy.

To many a reader, this might come as a surprise. Are pardons not granted by public officials, and should therefore be characterised as an exercise of public autonomy? I will explain below why this assumption is wrong, by pointing to the fact that executive mercy has historically not been a power of the state—that is, a public power in the narrow sense of public autonomy—but a power of an individual, the king, acting with private autonomy.^76^

Before I move on, however, it is important to emphasise that Sarat and Hussain’s argument about the ‘lawlessness’ of pardons is particularly relevant for our discussion of the Conflict Thesis. Although Sarat and Hussain do not themselves make the connection between their argument and the rule of law, conceptualising pardons as forms of ‘lawful lawlessness’ is a central piece in the puzzle that allows us to disprove the Conflict Thesis. The main reason for this is that once we see pardons as ‘lawful lawlessness’, they cannot be criticised for violating the rule of law—or any specific law, for that matter. Hence, if a state official decides to pardon prisoner A but not prisoner B, even if their cases are alike in all relevant respects, her decision cannot be criticised for violating the principle that like cases ought to be treated alike. This is because, first of all, the rule of law simply does not apply where legal rules have been abrogated. Secondly, private autonomy is not subject to the rule of law and its requirement to treat like cases alike.

However, while Sarat and Hussain’s theory can usefully be applied to resolve the Conflict Thesis, I believe that its framework—the Schmittian state of exception—clouds our view of the true nature of pardons. Specifically, I will argue in the following section that the fact that pardons suspend only isolated legal rules for the benefit of a person, rather than the whole juridical order, is essential to their function as last resorts. Pardons, I will propose, are thus best understood as miracles, not as states of exception.

### B. The Miracle of Mercy

To understand why pardons are best conceived of as miracles, we first need to shed light on why Sarat and Hussain’s analogy between pardons and the Schmittian state of exception fails. There are two important differences between the concept of lawful lawlessness as it applies to executive mercy and Schmitt’s theory of the state of exception: the first difference is that between the totality of the state of exception and the individuality of pardons, the second that between the neutrality of the state of exception and the partiality of pardons. I will explain these points in turn.

First, totality versus individuality. As outlined above, in Schmitt’s theory of sovereignty, the state of exception results in the ‘suspension of the legal order in its totality’, as Agamben puts it.^77^ Pardons, by contrast, only suspend isolated legal rules for an individual person. Executive mercy results in the suspension of specific parts of the law, such as the laws against murder, as applied in one particular case. Hence, even in cases of capital punishment, pardons are considerably less momentous than decisions on the state of exception, which affect the totality of the legal order.

Secondly, neutrality versus partiality. The state of exception in Schmitt’s sense is one that is decided by the sovereign on purely political grounds. Although it may serve specific political goals, it is not a decision whose declared aim it is to benefit a specific individual. Rather, as indicated above, the purpose of the state of exception is to thwart serious dangers that threaten the whole community, rather than just individual members of that community.^78^ In other words, the state of exception is neutral with respect to the persons who may end up benefiting from it.^79^ This is not so with pardons. In contrast to the state of exception, one of the constitutive features of pardons is that they benefit a person who is in an unequal position with the mercy-giver. Pardons are therefore partial in that they are aimed at benefiting only a specific person or specific persons.

For these reasons, Sarat and Hussain go astray when trying to illuminate pardon’s ‘alegality’ by drawing on Schmitt’s state of exception. Schmitt’s exception is a totalitarian one and does not have the purpose of benefiting anyone in particular. Pardons, by contrast, are sovereign exceptions only to individual legal rules and are aimed at serving specific people.

That is not to say, however, that Schmitt’s thinking is of no avail when it comes to understanding the nature of pardons. Schmitt was right when he famously emphasised that ‘concepts of the modern theory of the state are secularized theological concepts’.^80^ Although he only mentioned mercy in passing, he pointed out in *Political Theology* that because of its ability to suspend the normal course of things ‘the exception in jurisprudence is analogous to the miracle in theology’.^81^ Where an omnipotent God decides on miracles, an omnipotent secular sovereign decides on the exception. Schmitt returns to the dichotomy between the natural and the supernatural—between law and exception—in his later works. In *The* Nomos *of the Earth*, he argues that the appropriation of land ‘is the primary legal title that underlies all subsequent law’.^82^ All law, in other words, begins with the ordering of the land. And land, of course, is the epitome of nature. Its ordering, therefore, is the ordering of the *natural*. And as Schmitt explains in *On the Three Types of Juristic Thought*, the *supernatural* finds its representation in mercy, which is not part of ‘a humanized normativistic order’ but ‘belongs in an exalted Divine order above human normativization’.^83^ Mercy—belonging to the supernatural domain—cannot be ordered in the same way that law—ie the natural—can. Mercy has its source in the divine, law in nature. Order (law) is contrasted with the unorderable (mercy), nature with the supernatural. Mercy and law exist in separate worlds.

I propose that we can identify at least four striking similarities between mercy (and pardons in particular) and the divine miracle. First, in the case of theological miracles, God is considered to have complete discretion in His actions. He is by definition not bound by any rules because He is Himself the creator of those rules. If God effects a miracle, then He does so with absolute discretion. Likewise, pardons are a matter of the sovereign (ie executive body), who has absolute discretion. No sovereign is legally required to either grant a pardon or not. Secondly, by inference, no one has an entitlement or right to a miracle. In the same way, no one has an entitlement to be shown mercy through a pardon. As discussed above, neither mercy nor pardons are acts someone can demand. Thirdly, miracles are not something that can be deduced from the laws of nature for, while it is often said that the Christian God works *through* nature, a miracle in fact *defeats* nature. This is possible because, as nature’s creator, God can choose to work against nature. A divine miracle is thus something that was not supposed to happen according to the laws of nature. Pardons, by analogy, suspend the course of the laws of man. As argued above, they do so surgically, as it were, by setting aside only specific rules. If, for instance, the law demands that a person spends 20 years in prison, a pardon sets this legal command aside, while leaving the others intact. This is what it means to say that pardons are provided by but not regulated by the law, for what is set aside can no longer bind. Finally, a miracle is always a benevolent act. It is to the benefit of its recipient.^84^ As I have shown, the same holds true for pardons. They are partial in that they are always intended to benefit a specific individual or specific individuals.

Comparing pardons to theological miracles is not only interesting for its own sake, however. Most importantly, this comparison sheds light on the conceptual structure of pardons and, in doing so, allows us to discern why understanding pardons as miracles is more fruitful than following the more common interpretation of pardons as states of exception. As I will demonstrate in what follows, pardons *need* to have a ‘miraculous’ nature in order to fulfil their function as a last resort against legal decision making that has gone awry. Put differently, if pardons were not like miracles but instead like states of exception, or were not like either, they could not serve their purpose. To understand this, we need to consider why legal systems have pardons in the first place.

One of the central advantages of the introduction of general rules in legal systems was that these rules apply equally to any potential case that would arise. However, generality may lead to injustice when relevant differences within similar cases are disregarded. Even the most developed legal systems face this dilemma. As James Sterba notes, ‘Well-designed institutional or legal rules … usually can only render it highly probable that a morally [j]ust result occurs; they cannot infallibly guarantee that result’.^85^ In order to correct the most egregious injustices, a device was required to remedy this situation by allowing for individual exceptions from the application of the general rules.

Pardons are that device. They intervene when the application of legal rules fails to deliver humane results.^86^ Blackstone identified this attribute of pardons when he held that if no ‘other plea [] will avail to avoid the judgment … the *last and surest resort* is in the king’s most gracious pardon’.^87^ As a last resort, pardons provide an opportunity to correct an injustice that was caused by the legal system but cannot be solved by the legal system. As Carla Ann Hage Johnson emphasises, ‘The central notion behind this understanding of pardon is that law needs a mechanism for ensuring fairness, for allowing for individuation between cases where the universal law itself cannot’.^88^

The personal element in pardons, which we encountered above in our discussion of private autonomy, plays a focal role in the function of pardons as a last resort. Those endowed with pardon powers are not mere (legal) officials. They are always also human beings who are expected to act based on personal considerations.^89^ In virtue of this fact, they are believed to be able to give a humane touch to the system by correcting unjust or inconvenient legal outcomes. In a government of laws, there should always be a person with the power to intervene on humane grounds. As such, those who hold the power to show mercy contrast with judges, who, according to Murphy, are representatives of the rule of law, not their ‘own feeling[s]’.^90^

This detachment of the personal from the legal realm explains the prominent place of sovereignty in pardons. Rousseau made this very clear when he wrote that ‘The right of pardoning … belongs only to the authority which is superior to both judge and law, ie the Sovereign’.^91^ The king in particular was often regarded as uniquely suited for dispensing pardons. As Montesquieu observed, pardons are ‘the characteristic of monarchs’.^92^ The same idea can be found in Blackstone, who noted that ‘the great operation of [the King’s] sceptre is mercy’.^93^ Similarly, Jeremy Bentham, held that ‘so long as you have a king … you will have a functionary, in whose hands this same pardon-power will remain lodged’.^94^

That only the sovereign was regarded as being able to dispense mercy was not a coincidence. In fact, it is a conceptual necessity that the one who grants mercy stands outside the legal system. The reason for this is that only someone who is not part of the legal system can adopt the measures necessary to solve the problems created by the system itself. It is therefore more or less explicitly acknowledged in the writings referred to above that, sometimes, the only right thing to do is to suspend the normal course of (legal) affairs.

What the alternative to this would look like is expressed vividly in Pope Hadrian VI’s dictum *fiat iustitia et pereat mundus*: that justice (understood as the rigid application of the law) should take precedence even if the world would perish as a result.^95^ If one disagrees with this radical view—ie the view that the law must be applied regardless of its consequences—then, importantly, only an extra-legal device that is individual and partial in the way pardons are would be able to fulfil this role, to act as a corrective mechanism to concrete injustices. This is because the ‘outsider status’ of executive mercy exempts it from the scope of the legal system while at the same time allowing it to make surgical interventions in said system where necessary. Pardons are in this sense much like operators in automatic trains. They are not required when things run smoothly. However, in cases of emergency when the system fails, they are there to intervene in a system they are not technically a part of.

This exemption from specific legal rules is key precisely because it is these rules which have failed to provide justice in the particular case. Importantly, this is not a failure of the law in the strict sense. Rather, it is simply the law’s natural limitation. Law could never do the job for which pardons have been created for. Rachel Barkow makes a similar point when observing that, if we were to apply the logic of law, we would expect

some specified standard to allow good reasons for [pardons] to be sifted from bad ones. But ex ante specification of when mercy is appropriate contradicts the reason for having mercy in the first place: it is because not all factors can be anticipated in advance that the discretionary check is important.^96^

To have the law dictate the terms of pardons would thus be to put the fox in charge of the henhouse. That is not to say, however, that the legal system should not aspire to be as just to the individual case as possible. This was emphasised by the Italian legal thinker Cesare Beccaria, who held that ‘Clemency is a virtue which belongs to the legislator, and not to the executor of the laws, a virtue which ought to shine in the code, and not in private judgment’.^97^ Beccaria’s point is that the more just a state is, the less need it has for pardons. For a state to become more just, he proposed, it should leave to the legislator the considerations that underlie executive mercy, that is, to ‘be tender, indulgent, and humane’.^98^ Beccaria’s ideal—unachievable, but still worth striving for—was a state that does not require pardons because its laws are sufficiently just and kind-hearted.^99^

This ideal of a pardon-free state also explains the difficult relationship between the rule of law and executive mercy. As I argued above, the conceptual nature of pardons—a last resort against legal decision making that has gone awry—implies that they are not based on any rules as to whether to grant them or not. It also implies that they should not be subject to judicial review. This is because, in attempting to review an official’s decision (not) to pardon someone, a court would have to be able to find a set of rules or standards which would allow it to assess whether or not that decision was correct. However, the precise point of pardons is that there are no such rules or standards, and that there should not be any.^100^ Nevertheless, as I will show in the next section, many jurisdictions have been trying to rein in pardon powers by subjecting them to the rule of law.

### C. The Demise of the Miracle?

In the past few decades, pardon powers have come under increased scrutiny in legal systems around the world. Dissatisfied with pardons’ aversion to being governed by rules, many jurisdictions have called on the rule of law to subject the exercise of pardon powers to legal assessment—with quite some success.^101^ For example, as Adam Perry and Andrew Novak have independently pointed out, while, in the common law, the prerogative of mercy used to be ‘free from legal constraint’^102^ and therefore ‘outside the reach of judicial inquiry’,^103^ courts have become increasingly willing to review pardon decisions. In fact, this seems to have become a trend in recent times.^104^ This is because acts that are not supposed to be reviewed legally have become more and more suspect in modern states. As Barkow notes, ‘With the rise of administrative law, our legal culture has come to view unreviewable discretion to decide individual cases as the very definition of lawlessness’.^105^ In particular, pardons, she argues, ‘sit uneasily beside an administrative state that faces such scrutiny, for these exercises of mercy are precisely the type of unreviewable exercises of discretion that administrative law seeks to control’.^106^

This tendency of law to regulate previously unregulated domains is part of what Lord Sumption has recently called ‘Law’s Expanding Empire’.^107^ The law, Sumption argues, has an expansionist tendency: it ‘penetrates every corner of human life’.^108^ This means that the law is taking over the space once occupied by politics, as well as the space that ‘once belonged exclusively to the domain of personal judgment’.^109^ As he elaborates, ‘the special areas that were once thought to be outside the purview of the courts … have all one by one yielded to the power of judges’.^110^ The cause Sumption diagnoses for this trend is the diminished degree of risk that people are willing to take: ‘we are no longer willing to accept the wheel of fortune as an ordinary incident of human existence’.^111^ Although intensified in recent years, law’s tendency to expand, and to rule in previously unruled domains, is not a new phenomenon. Schmitt, in *Political Theology*, had already bemoaned the ‘tendency of liberal constitutionalism to regulate the state of exception as thoroughly as possible’.^112^ To be sure, Schmitt’s is a more limited point than Sumption’s, but they are diagnosing a similar issue.

The increasing willingness of some jurisdictions to subject pardon decisions to judicial review raises the question of whether we can still say that pardons are miracles. Recall that I explained the miracle of mercy by showing that pardons are an essentially lawless occurrence. Pardons, I said, are like a gift. They are an act of grace that cannot be subject to legal norms, for if they could, they would not be able to fulfil their purpose of stepping in when these legal norms fail. So can pardons be subject to judicial review while still remaining miracles? Or is this the demise of the miracle of mercy?

Yes and no. First, consider the sense in which the development to legally constrain pardons does not affect their character as miracles. It is important to note that the extent to which pardons have been made the subject of judicial review is, in fact, quite limited. The courts that have broken with the sacrosanct nature of executive mercy, for the most part, only scrutinise mercy *proceedings*; they stop short of reviewing the ultimate pardon decision itself.^113^ In other words, they allow for procedural review as opposed to substantive review. For example, some courts have imposed (minimum) due process standards, such as a duty to listen to the mercy-seeker’s arguments. Or they have established a more general right to a fair hearing.^114^ However, the official’s personal decision to grant or refuse a pardon request itself has mostly remained unchallenged by the courts. The bottom line is therefore that while courts allow for some limited judicial review with respect to the pardon proceedings, they generally accept that individual pardons should not be reviewed on their merits.^115^

Assume, however, that courts started subjecting pardon decisions to substantive review. For instance, courts could impose substantive rules as to what constitutes an invalid reason for granting (or refusing to grant) mercy, such as a ban on pardons that discriminate on the basis of race. Some courts, in fact, have already indicated that they are open to subjecting pardons to such substantive review,^116^ or have done so already.^117^ Would this not show that the days of mercy’s miracle are numbered?

To understand why this is not the case, recall the distinction I drew above between the two different aspects of executive mercy: it abrogate individual legal rules and it is discretionary. Attempts to review pardon decisions substantively only affect the latter aspect, not the former. Put differently, while legal restrictions limit the scope of officials’ pardon powers, they do not alter one of the miraculous effects of pardon decisions, that is, their setting aside of individual laws in specific cases. This lawlessness or alegality remains, even if its domain becomes more limited. What the partial subjection to judicial review thus does is diminish the ‘room’ for pardons understood as an act of grace—freely given as the sovereign wishes.

However, it would be a mistake to believe that pardon powers remain completely unaffected by their subjection to legal constraints. As the distinction between the two aspects of pardons makes clear, there is one aspect—the aspect of discretion—which does become compromised. By contrast to the first aspect of pardons, the discretionary element involved in pardons is very much a question of degrees. The greater the constraints are that judges impose on pardon powers, the smaller the scope of discretion that remains for those powers and the weaker the miracle of mercy becomes.

Perhaps a metaphor will serve to sum up the main insights of this section. Mercy, we might say, is much like a plant. The organic makeup of a plant remains the same, regardless of its size. The same holds for pardons, which retain their ability to set aside laws regardless of circumstances. But a plant can come in different sizes—some mere sprouts, others majestic trees. The smaller and more clipped they are, the less space they take up and the more room there is for other things. Similarly, pardons can be trimmed to different degrees. The more legal constraints are imposed on them, the less powerful these powers are and the more limited the degree to which they can work their miracle.

## 4. Conclusion

I started this article with the puzzling case of the two Damper brothers from Mississippi who asked the president for mercy. Even though their cases were alike, they were treated differently. One was pardoned, the other was not. I dedicated this article to analysing and resolving the underlying issue of the brothers’ case.

As I have argued, one of the central principles of the rule of law is the principle that like cases be treated alike. Mercy, on the other hand, is believed to be like a gift whose giver is not constrained by any such principles. Hence, if we required mercy-givers—ie state officials with pardon powers like presidents—to apply mercy equally in all equal cases, then they would cease to be *mercy*-givers. However, as I have demonstrated, state officials with pardon powers need to be *true* mercy-givers so that pardons can fulfil their function: to correct undesirable outcomes yielded by the normal application of the law.

I have proposed that this conflict can be resolved—that is, the Conflict Thesis can be disproved—if we understand mercy as a miracle. Pardons in particular, I argued, are like miracles: they are the benevolent and discretionary suspension of individual laws by a greater power. In other words, pardons set aside the law, which makes them an essentially lawless occurrence. As a consequence, executive mercy is an act that cannot be evaluated against the rule of law. This is because once the law has been set aside, it no longer applies to the given situation, and the conflict between pardons and the rule of law’s requirement to treat like cases alike disappears.

## Footnotes

1 Gregory Korte, ‘Two Brothers, Two Petitions for Clemency, Two Different Outcomes’ *USA Today* (9 January 2017) <https://eu.usatoday.com/story/news/politics/2017/01/09/two-brothers-two-petitions-clemency-two-different-outcomes/96297020/> accessed 1 December 2020; petition for clemency denied in the case of Harold Damper, see ‘Commutations Denied by President Barack Obama’ <www.justice.gov/pardon/obama-denials/commutations-denied-president-barack-obama> accessed 1 December 2020; petition for clemency granted in the case of Dewayne Damper, see ‘Commutations Granted by President Barack Obama (2009-2017)’ <www.supremecourt.gov/opinions/URLs_Cited/OT2017/16-1423/16-1423-1.pdf> accessed 1 December 2020.

2 What a ‘relevant respect’ is (and, equally important, what it is not) is something one has to find out by interpreting the law. Note also that I am simplifying the facts of the two brothers’ cases for the sake of the argument.

3 See eg Andrew Novak, *Comparative Executive Clemency: The Constitutional Pardon Power and the Prerogative of Mercy in Global Perspective* (Routledge 2017) 3, who notes that ‘Discretionary executive clemency sits uncomfortably in a legal system of objective rules and distinctions, and the pardon power has triggered different philosophical objections’; Austin Sarat, ‘At the Boundaries of Law: Executive Clemency, Sovereign Prerogative, and the Dilemma of American Legality’ (2005) 57 American Quarterly 611.

4 I am not ruling out that there are conceptions according to which the rule of law is inherently connected to questions of justice. However, even on those conceptions, the distinction between the two versions of the treat like cases alike principle does not necessarily have to be abandoned. See also s 2A below. I thus take the view in this article that the rule of law and justice are two distinct concepts that can be in conflict with each other. A similar question arises with respect to mercy and justice: see n 31.

5 Alex Tuckness and John M Parrish, *The Decline of Mercy in Public Life* (CUP 2014) 252.

6 ibid 253.

7 ibid 254.

8 Paul Twambley, ‘Mercy and Forgiveness’ (1976) 36 Analysis 84, 87.

9 William Shakespeare, *The Merchant of Venice* (Jay L Halio ed, OUP 1993) Act IV, scene 1; Jeffrie G Murphy, ‘Mercy and Legal Justice’ (1986) 4 Social Philosophy and Policy 1, 2–3.

10 Murphy (n 9) 3.

11 Ned Markosian, ‘Two Puzzles about Mercy’ (2013) 63 Philosophical Quarterly 269, 272.

12 Adam Perry, ‘Mercy’ (2018) 46 Philosophy & Public Affairs 60, 68; Murphy (n 9) 3.

13 At least if we follow correlativity theories, such as Wesley Hohfeld’s, according to which (claim) rights and duties are correlative: Wesley Newcomb Hohfeld, ‘Some Fundamental Legal Conceptions As Applied in Judicial Reasoning’ (1913) 23 Yale LJ 16, 30.

14 For a similar point, see Daniel Statman, ‘Doing Without Mercy’ (1994) XXXII Southern Journal of Philosophy 331, 334.

15 What makes cases alike is inherent in the rule itself. Kenneth Winston makes this point when he suggests that like cases ‘are cases subsumable under the same rule of law, ie, cases satisfying an identical legal description’: Kenneth I Winston, ‘On Treating Like Cases Alike’ (1974) 62 CLR 1, 16.

16 This principle is not to be confused with a similar principle that requires the alike treatment of like cases arising from justice. I take these principles to be distinct and focus on the rule of law principle in this article.

17 Brian Z Tamanaha, *On the Rule of Law: History, Politics, Theory* (CUP 2012) 144.

18 ibid 119.

19 ibid.

20 Brian Z Tamanaha, ‘A Concise Guide to the Rule of Law’ in Gianluigi Palombella and Neil Walker (eds),*Relocating the Rule of Law* (Hart Publishing 2009) 3–16,10.

21 Lon L Fuller, *The Morality of Law* (revised edn, Yale UP 1969) 39.

22 ibid 81.

23 Andrei Marmor, ‘The Rule of Law and Its Limits’ (2004) 23 Law and Philosophy 1, 6–7, 34–8. Note that Marmor has doubts about whether the ‘*desired* level of congruence between the law and its application’ must be a ‘perfect match’ (ibid 35, original emphasis). Marmor, inspired by Kelsen, distinguishes between two sets of rules: one addressed to the public and one addressed to officials. As he points out, ‘generally they ought to be identical’ (ibid 38). However, he goes on to argue that ‘some discrepancy between the content of the rules addressed to the public and those addressed to officials is not always regrettable’ in order ‘to accommodate conflicting purposes’ (ibid 37), which means that certain exceptions to the principle of congruence are justified (ibid 38).

24 ibid 34.

25 Ben Johnson and Richard Jordan, ‘Should Like Cases Be Decided Alike?: A Formal Analysis of Four Theories of Justice’ (2018) 18 <https://ssrn.com/abstract=3127737>.

26 Tamanaha (n 17) 119. Emphasis added.

27 Winston (n 15) 16.

28 HLA Hart, ‘Positivism and the Separation of Law and Morals’ (1958) 71 Harv L Rev 593, 623–4.

29 HLA Hart, *The Concept of Law* (3rd edn, OUP 2012) 206.

30 Anselm of Canterbury, *Anselm of Canterbury: The Major Works* (Brian Davies and GR Evans eds, OUP 1998) ch 11.

31 It is worth noting that, according to Anselm’s theory, mercy and justice are two distinct virtues that can in principle conflict with each other. While not everyone in the literature agrees with this view, I follow Anselm in drawing a distinction between these two virtues. This view has recently also been defended by Malcolm Bull, who has argued convincingly that mercy and justice are separate virtues, applicable in different circumstances and characterised by different requirements. Malcolm Bull, *On Mercy* (Princeton UP 2019). See eg p 73, where Bull writes that ‘mercy seems applicable in many cases in which justice is not’.

32 Ross Harrison, ‘The Equality of Mercy’ in Hyman Gross and Ross Harrison (eds), *Jurisprudence: Cambridge Essays* (Clarendon Press 1992).

33 ibid 107.

34 ibid.

35 ibid.

36 ibid.

37 ibid.

38 ibid.

39 ibid.

40 ibid.

41 ibid.

42 ibid 107–8.

43 ibid 108.

44 ibid 117.

45 Anselm of Canterbury (n 30) 93.

46 Harrison (n 32) 118.

47 ibid 117, 118.

48 I understand ‘sovereign’ in a loose sense here that is not limited to the Schmittian definition of the sovereign.

49 In some states, the parliament decides upon applications for pardons. This is the case, for example, in Switzerland, where the National Council (House of Representatives) together with the Council of States (Senate) decide as the United Federal Assembly on applications for pardons in a joint proceeding: Federal Constitution of the Swiss Confederation of 18 April 1999, art 157(1)(c).

50 A pardon, Carolyn Strange points out, ‘mitigates or annuls sanctions imposed through judicial findings, from minor fines to the death penalty’. Carolyn Strange, ‘Mercy and Parole in Anglo-American Criminal Justice Systems from the Eighteenth Century to the Twenty-First Century’ in Paul Knepper and Anja Johansen (eds), *The Oxford Handbook of the History of Crime and Criminal Justice* (OUP 2016) 573.

51 Pardons as understood in this article are different from other legal instruments that sometimes go by the name of ‘pardon’. For instance, several US states provide for so-called ‘innocence’ (eg North Carolina) or ‘absolute’ (eg Virginia) pardons, which are both reserved for persons who were wrongfully convicted <https://ccresourcecenter.org/state-restoration-profiles/50-state-comparisoncharacteristics-of-pardon-authorities-2/> accessed 15 January 2021. However, in contrast to how I define pardons in this article, these ‘pardons’ are usually understood to neither be completely discretionary nor to set aside the law. In the case of actual innocence, the law is not set aside by absolute or innocence ‘pardons’ because, as the person was wrongfully convicted, the law in question never applied to that person in the first place. Furthermore, there are legal instruments, such as the so-called compassionate release, which bear resemblance to pardons in that they result in the release of a convicted person before they have served their sentence. However, compassionate release ‘pardons’ are usually granted in response to serious illness or other unforeseen circumstances and are often necessitated by a lack of adequate care within the prison system. The more limited set of (humanitarian) reasons that can justify compassionate release suggest that, in comparison to pardons, rule of law principles may be applicable to this instrument. Pardons can also be distinguished from so-called legislative amnesty. Not only are amnesties granted by a different actor (parliament instead of the executive); they also apply in different situations. One difference is that while a pardon is ‘surgical’ in that it pertains to one specific individual only, amnesties generally apply to a selected group of people. Furthermore, amnesties are granted by the legislature, which is constrained by the constitution. They are not a matter of ‘private autonomy’.

52 A sceptic may object that pardons only suspend the law’s consequences rather than actually abrogating it. In the case of pardons, the reasoning goes, the legal rules apply; it is just that they are not given any effect. This objection ignores that it is of the essence of legal rules that they lay claim to changing our normative landscapes. Once officials deny that claim, they deny the legal character of these rules. For this reason, I hold that the suspension of the law’s effects is tantamount to an abrogation of the law.

53 For the UK, see ‘Prerogative of Mercy’ in Jonathan Law and Elizabeth A Martin, *A Dictionary of Law* (7th edn, OUP 2014). The prerogative of mercy is defined as ‘The power of the Crown, on the Home Secretary’s advice, to pardon a criminal offence absolutely (and thereby relieving the defendant of all the consequences of conviction), to commute a sentence to a milder form, or to remit a sentence in part’.

54 RS Downie, ‘Forgiveness’ (1965) 15 Philosophical Quarterly 128, 131.

55 Austin Sarat and Nasser Hussain, ‘On Lawful Lawlessness: George Ryan, Executive Clemency, and the Rhetoric of Sparing Life’ (2004) 56 Stan L Rev 1307, 1314.

56 Carl Schmitt, *Political Theology: Four Chapters on the Concept of Sovereignty* (The MIT Press 1985) 5; Giorgio Agamben, ‘The State of Emergency’ (Centre Roland-Barthes (Université Paris VII, Denis-Diderot) 2002) <www.generation-online.org/p/fpagambenschmitt.htm>.

57 See, however, Schmitt, *Political Theology* (n 56) 12–13 (arguing that ‘The exception remains, nevertheless, accessible to jurisprudence because both elements, the norm as well as the decision, remain within the framework of the juristic’). The state of exception in Schmitt’s sense actually involves two kinds of decisions: the decision to suspend the law and the decisions the sovereign takes once the law has been suspended (I am grateful to Lars Vinx for this point). In pardons, this distinction is not as clear-cut, which is perhaps why Sarat and Hussain do not draw it at all. Because my argument is developed in response to theirs, I will follow their reading here.

58 Giorgio Agamben, *Homo Sacer: Sovereign Power and Bare Life* (Stanford UP 1998) 170.

59 Sarat and Hussain (n 55) 1312.

60 Schmitt, *Political Theology* (n 56) 7.

61 ibid 6.

62 ibid 6–7.

63 Sarat and Hussain (n 55) 1311.

64 ibid 1312.

65 See also Linda Ross Meyer, who resorts to describing pardon power as a ‘hole in the law’. Linda Ross Meyer, ‘The Merciful State’ in Austin Sarat and Nasser Hussain (eds), *Forgiveness, Mercy, and Clemency* (Stanford UP 2007) 85.

66 David Dyzenhaus, *The Constitution of Law: Legality in a Time of Emergency* (CUP 2006) 35–54.

67 See also Ross Meyer (n 65) 64 (noting that ‘the executive’s pardon power is ungoverned by rule of law’).

68 Sarat and Hussain (n 55) 1313, fn 30. Sarat and Hussain focus on executive clemency in cases of capital punishment. The reason why these cases may appear to be particularly interesting is that they involve high stakes. As Sarat and Hussain point out, the decision over life and death is the constituent condition of ancient and contemporary sovereignty, that is, the sovereign right to kill and its corollary, the ‘right to spare life’, by pardoning those who were sentenced to death. While these high stakes make certain pardon cases particularly interesting, nothing essential turns on whether or not the decision concerns a matter of life or death. The more important point is that what pardons entail is a fundamental decision as to whether or not the law applies to a given case or not.

69 Hart (n 29) 81.

70 ibid.

71 Alf Ross, ‘On the Concepts “State” and “State Organs” in Constitutional Law’ (1961) 5 Scandinavian Studies in Law 111, 117; Hart distinguishes between public and private power, Hart (n 29) 81.

72 Ross (n 71) 117.

73 ibid 118.

74 Philip Mullock, ‘Power-Conferring Rules & the Rule of Recognition’ (1974) 36 U Pitt L Rev 23, 28.

75 Ross (n 71) 118.

76 As Chief Justice Marshall put it: a pardon ‘is the private, though official, act of the executive magistrate’ *United States v Wilson* 32 US 150 (1833) 160.

77 Agamben (n 56).

78 As Sarat and Hussain themselves acknowledge, pardons usually do not ‘deal in terms of imminent peril or collapse; its usual idiom is one of mercy and not danger’. Sarat and Hussain (n 55) 1314.

79 Except, of course, for those whom the sovereign considers enemies.

80 Schmitt, *Political Theology* (n 56) 36.

81 ibid.

82 Carl Schmitt, *The* Nomos *of the Earth in the International Law of the Jus Publicum Europaeum* (GL Ulmen tr, Telos Press Publishing 2006) 46.

83 Carl Schmitt, *On the Three Types of Juristic Thought* (Joseph W Bendersky tr, Praeger Publishers 2004) 61.

84 Even if, as for example in the case of the Ten Plagues, this benefit comes at a certain cost.

85 James Sterba, ‘Can a Person Deserve Mercy?’ (1979) 10 Journal of Social Philosophy 11, 11.

86 See Ross Meyer (n 65), who argues for the power of the executive to pardon, that is, for a ‘merciful state’ on grounds that we need pardons to bring about justice when the law is too harsh.

87 William Blackstone, *Commentaries on the Laws of England, Vol IV* (1765) 389. Emphasis added.

88 Carla Ann Hage Johnson, ‘Entitled to Clemency: Mercy in the Criminal Law’ (1991) 10 Law and Philosophy 109, 115.

89 This feature is not as rare as it might seem at first glance. In particular, pardons resemble other royal prerogative powers at common law, in particular the power to confer honours. Just like mercy, the granting of a title of honour is a personal gift bestowed by the sovereign on one of her subjects. This power to grant honours is also considered to be a personal prerogative because it entails—at least with respect to some royal honours—‘the personal involvement of the Sovereign’. Noel Cox, *The Royal Prerogative and Constitutional Law: A Search for the Quintessence of Executive Power* (Routledge 2020) 78. The personal element is not the only similarity between mercy and the prerogative to confer honours. As with the legally unlimited power to grant mercy, ‘The Sovereign is legally and practically the sole fount of titles and awards of honour and dignity … The powers of the Crown in this respect are unlimited’ (ibid 70).

90 Jeffrie G Murphy and Jean Hampton, *Mercy and Forgiveness* (CUP 1998) 174.

91 Jean Jacques Rousseau, *The Social Contract and Discourses* (GDH Cole tr, JM Dent and Sons 1923) 31 (emphasis removed).

92 Charles Louis de Secondat Baron de Montesquieu, *The Complete Works of M de Montesquieu in Four Volumes, Vol 1. The Spirit of Laws* [1748] (T Evans 1777) Book VI, ch XXI.

93 Blackstone (n 87) 389.

94 Jeremy Bentham, *The Works of Jeremy Bentham, Vol I. Principles of Morals and Legislation, Fragment on Government, Civil Code, Penal Law* (John Bowring ed, William Tait 1838) Appendix ch V–VI, para 6.

95 Note that this is only one of several possible interpretations of this maxim. See Detlef Liebs, ‘The Legal Proverb: Fiat Iustitia et Pereat Mundus. Das Rechtssprichwort: Fiat Iustitia et Pereat Mundus’ (2017) 4 JOSHA 1.

96 Rachel E Barkow, ‘The Ascent of the Administrative State and the Demise of Mercy’ (2008) 121 Harv L Rev 1332, 1364.

97 Cesare Beccaria, *An Essay on Crimes and Punishments* (Alexander Donaldson 1778) 167.

98 ibid 167–8; see also Kathleen Dean Moore, *Pardons: Justice, Mercy, and the Public Interest* (OUP 1989) 39.

99 Moore (n 98) 39, 76.

100 Barkow (n 96) 1363–4.

101 There is not enough space here to analyse this tendency in any depth, which is why I will limit myself to highlighting some of the central cases that have tried to rein in pardon powers. To the best of my knowledge, India’s case *Maru Ram v Union of India* AIR 1980 SC 2147 can be considered to be the forerunner, with other states to follow suit (for an analysis of the development in the case law of Indian courts, see Adam Perry, ‘Mercy and Caprice under the Indian Constitution’ (2017) 1 Indian Law Rev 56). Particularly noteworthy is the Commonwealth case law on the amenability to judicial review of the exercise of the prerogative of mercy. One important example is *De Freitas v Benny* (1976) AC 234 (PC), a case that originated in Trinidad and Tobago. In this case, the Privy Council stated that ‘mercy is not the subject of legal rights. It begins where legal rights end.’ As a result, it refused to review a denial of a pardon in a death penalty case. This decision was confirmed in *Reckley v Minister of Public Safety and Immigration (No 1)* (1996) AC 527 (PC), a case arising in the Bahamas. The Privy Council changed its mind two decades later in *Lewis v Attorney General of Jamaica* (2000) UKPC 35. In this case, it overruled its previous case law by holding that the applicant’s constitutional rights were violated as he was not granted a fair hearing in the process relating to his petition for clemency. Here, the Privy Council thus held that exercises of the prerogative of mercy are subject to judicial review on procedural grounds. Last but not least, in *Pitman v The State* (2017) UKPC 6 the Privy Council finally laid the ground for judicial review of pardon decisions on substantive grounds.

102 Adam Perry, ‘Who’s Afraid of Mercy?’ (*Judicial Power Project*, 5 February 2016) <https://judicialpowerproject.org.uk/whos-afraid-of-mercy/> accessed 1 December 2020.

103 Novak (n 3) 170.

104 Adam Perry, ‘Mercy and Judicial Review in the Commonwealth’ (*ICONnect: Blog of the International Journal of Constitutional Law*, 2 February 2015) <www.iconnectblog.com/2015/ 02/mercy-and-judicial-review-in-the-commonwealth/>; see also Novak (n 3) ch 8.

105 Barkow (n 96) 1335.

106 ibid.

107 BBC Radio 4, ‘Law’s Expanding Empire’, *The Reith Lectures* (25 May 2019) <www.bbc.co.uk/programmes/m00057m8> accessed 1 December 2020.

108 ibid minute 8:03.

109 BBC Radio 4 (n 107).

110 ibid minute 9:35.

111 ibid minute 28:37.

112 Schmitt, *Political Theology* (n 56) 14.

113 Novak (n 3) 194.

114 Australia is an example. See ibid 177–8. See also *Eastman v Attorney-General for the Australian Capital Territory* [2007] ACTSC 28 (29 January 2007); *Edwards v Santos Limited* [2011] HCA 8 (30 March 2011).

115 For an in-depth analysis of the case law in common law nations, see Novak (n 3) ch 8.

116 See eg *Pitman* (n 101).

117 South Africa is a case in point. See Karthy Govender, ‘Judicial Review of the Pardon Power in Section 82(2)(J) of the Constitution of the Republic of South Africa’ (1996) 23 Stellenbosch Law Review 490.

